# The role of platelet-related parameters for the prediction of NAFLD in OSAHS patients

**DOI:** 10.1186/s12890-022-02291-6

**Published:** 2022-12-24

**Authors:** Menglan Chen, Biying Wang, Jiefeng Huang, Jianming Zhao, Jia Chen, Gongping Chen

**Affiliations:** 1 Department of Respiratory and Critical Care Medicine, The First Affiliated Hospital of Fujian Medical University, No. 20, Chazhong Road, Taijiang District, Fuzhou, 350005 Fujian Province People’s Republic of China; 2 Fujian Provincial Sleep-Disordered Breathing Clinic Center, No. 20, Chazhong Road, Taijiang District, Fuzhou, 350005 Fujian Province People’s Republic of China; 3 Institute of Respiratory Disease, Fujian Medical University, No. 20, Chazhong Road, Taijiang District, Fuzhou, 350005 Fujian Province People’s Republic of China; 4 Department of Respiratory and Critical Care Medicine, National Regional Medical Center, Binhai Campus of the First Affiliated Hospital, Fujian Medical University, Fuzhou, 350212 Fujian Province People’s Republic of China

**Keywords:** Obstructive sleep apnea–hypopnea syndrome, Non-alcoholic fatty liver disease, Platelet to lymphocyte count ratio, White blood cell count to mean platelet volume ratio

## Abstract

**Purpose:**

As the detection of non-alcoholic fatty liver disease (NAFLD) is imperative for the prevention of its complications, we aimed to explore the predictive value of platelet to lymphocyte count ratio (PLR) and white blood cell count to mean platelet volume ratio (WBC/MPV) in relation to the occurrence of NAFLD among patients with obstructive sleep apnea–hypopnea syndrome (OSAHS).

**Methods:**

This was a cross-sectional study consisting of 351 patients with OSAHS (279 with and 72 without NAFLD). The logistic regression analysis was performed to estimate associations between PLR, WBC/MPV, and NAFLD. Finally, the receiver operating characteristic curve (ROC curve) was used to analyze the efficacy of PLR and WBC/MPV in NAFLD prediction.

**Results:**

Compared to the OSAHS-only group, there was a rising trend in AHI and TS90% in the OSAHS + NAFLD group. And the logistic regression analysis identified average oxygen saturation (MaSO_2_), WBC/MPV and PLR as predicted factors (odds ratio [OR] = 1.134, *P* = 0.031; OR = 7.559, *P* = 0.018, OR = 0.980, *P* < 0.001, respectively) for NAFLD in OSAHS patients. Moreover, compared with WBC/MPV, PLR, FLI, and APRI, a combination of WBC/MPV and PLR presented the largest AUC for the detection of NAFLD in BMI < 28 kg/m^2^ (0.753, 95% CI 0.684–0.822), and in age ≥ 60 years subgroup (0.786, 95% CI 0.692–0.880) in ROC analysis. Meanwhile, a combination of WBC/MPV and PLR presented the second largest AUC for the detection of NAFLD in all subjects (0.743, 95% CI 0.708–0.831), as well as in the age < 60 years subgroup (0.729, 95% CI 0.652–0.806), only ranked after FLI, suggesting the combination of WBC/MPV and PLR has a good predictive value for NAFLD in OSAHS patients.

**Conclusion:**

We confirmed that the levels of WBC/MPV, PLR, and MaSO_2_ were closely related to the occurrence of NAFLD among OSAHS patients. Furthermore, our results highlighted the clinical combination of WBC/MPV and PLR levels could act as a simple and effective biomarker for screening NAFLD in patients with OSAHS.

## Introduction

Non-alcoholic fatty liver disease (NAFLD) is a spectrum ranging from non-alcoholic fatty liver (NAFL), to non-alcoholic steatohepatitis (NASH), to cirrhosis [1]. NAFLD has become the most common chronic liver disorder, with a global prevalence of 25.24% of the adult population, and the incidence is rapidly increasing year by year [2]. It was supposed that NAFLD would become the primary indication for liver transplantation in the future [3].

There is a growing body of evidence demonstrating a positive correlation between NAFLD and obstructive sleep apnea–hypopnea syndrome (OSAHS) [4, 5]. Of note, NAFLD is typically asymptomatic until the advanced stages, and the routine detection of NAFLD based on liver biopsy, sonography, liver/spleen CT (liver/spleen computed tomography), and magnetic resonance imaging (MRI) may not be feasible with respect to health care expenditures and biopsy-related risks [6], highlighting the importance of exploring accurate non-invasive tools for prediction and early diagnosis of fatty liver, particularly in high-risk groups including those with OSAHS.

A series of studies have shown that NAFLD was closely linked to subclinical inflammation [7, 8], and routine blood cells, including platelet and white blood cell, have proven to be good biomarkers for systemic inflammation [9, 10]. Thus, we tested the performance of the platelet-related parameters in the prediction of NAFLD.

Platelet to lymphocyte count ratio (PLR) is a novel biomarker that has been initially proposed as an ideal indicator of systemic inflammation [11–13]. Recently, emerging evidence indicated strong link in between OSAHS, liver diseases (including HBV/HCV and HCC), and PLR [14–17]. However, there was a lack of data regarding the association between PLR and NAFLD. White blood cell count to mean platelet volume ratio (WBC/MPV) was previously used as a predictor of thrombosis in cardiovascular and cerebrovascular illnesses [18–20]. However, there has been no study on the relationship between WBC/MPV, OSAHS, and NAFLD. Since OSAHS and NAFLD might both create pathological changes in the blood system, there was a possibility that WBC/MPV had a relationship with OSAHS and/or NAFLD. Furthermore, previous studies have confirmed non-invasive scores, such as the aspartate aminotransferase to platelet ratio index (APRI), and the fatty liver index (FLI) had good performance in assessing NAFLD [21, 22]. Accordingly, the present study aimed to evaluate the predictive power of platelet-related parameters (PLR and WBC/MPV) for detecting NAFLD in individuals with OSAHS compared with APRI and FLI.

## Material and methods

### Participants

The study cohort comprised individuals who attended our sleep center because of snoring, excessive daytime sleepiness, or other related symptoms, and were finally diagnosed with OSAHS by polysomnography between January 2016 and December 2020. The exclusion criteria included the following: (1) chronic obstructive pulmonary disease, bronchial asthma, interstitial lung disease, and other chronic lung diseases; and (2) severe heart failure, stroke, and other severe cardiovascular and cerebrovascular diseases; and (3) malignant tumor or major physical or mental illness; and (4) hepatic diseases such as viral hepatitis, autoimmune liver disease, and drug-induced liver disease; and (5) immune or hematological diseases such as rheumatoid arthritis; (6) acute and chronic infections; and (7) anti-platelet drugs used in the last month; and (8) excessive drinking (the amount of alcohol consumed in men is equivalent to > 30 g of ethanol per day, and is > 20 g/d in women); and (9) other sleep disorders, such as narcolepsy and restless leg syndrome, etc.; and (10) sleep apnea attributable to other causes, such as hypothyroidism and central and mixed sleep apnea; and (11) history of previous CPAP treatment; and (12) history of previous splenectomy. The study was approved by the First Affiliated Hospital of Fujian Medical University (Fuzhou, China) and all participants gave their written informed consent after a full explanation.

### Clinical data collection

All participants completed a detailed questionnaire on history of smoking and alcohol consumption, medical history (hypertension, diabetes mellitus, hyperlipidemia etc.), and medications, and physical examination. Anthropometric measurements including height and body weight were obtained with the participants wearing light clothing. The body mass index (BMI) was calculated as body weight/ height squared (kg/m^2^). Waist circumference (midway between the lower costal margin and iliac crest) and neck circumference (at the level of the laryngeal prominence) were measured with a tape. In addition, every subject completed an Epworth sleepiness scale (ESS) using a well-validated Chinese version with the score ranging from 0 to 24, and it was considered daytime sleepiness when ESS score was ≥ 10 [23].

### Laboratory measurements

Venous blood was taken the morning after polysomnographic evaluation. Blood routine, liver function, triglyceride (TG), total cholesterol (TC), and fasting glucose were tested in all patients. Blood routine was detected by Hitachi H-7600 autoanalyzer produced by Japan Co., Ltd., and blood biochemistry was performed using a Modular P800 autoanalyzer (Roche, Tokyo, Japan). PLR measured platelet count to lymphocyte count, and WBC/MPV measured white blood cell count to mean platelet volume. Platelet-related parameters including platelet count (PC), mean platelet volume (MPV), platelet distribution width (PDW), plateletcrit (PCT), average concentration of platelet contents (MPC) were also assessed. FLI and APRI were calculated using the following formulas [21, 22]: FLI = (e^0.953×ln(triglycerides)+0.139×BMI+0.718×ln(GGT)+0.053×waistcircumference−15.745^)/(1 + e^0.953×ln(triglycerides)+0.139×BMI+0.718×ln(GGT)+0.053×waistcircumference−15.745^) × 100 (with TG measured in mmol/l, GGT in U/l, and waist circumference in cm), APRI = (AST/upper limit of normal)/platelets (10^9^/L) × 100.

### Polysomnographic evaluation

Overnight polysomnography (P Series Sleep System, Compumedics, Melbourne, Australia) was conducted to diagnose OSAHS. Electroencephalography, electrooculography, electromyography, and electrocardiography were performed during polysomnography, and the variables assessed being as follows: oronasal airflow, thoracic and abdominal respiratory efforts, and pulse oxygen saturation. AHI was defined as the number of episodes of apnea and hypopnea per hour of sleep. ODI was defined as number of episodes of oxyhemoglobin desaturation ≥ 3% from the immediate baseline per hour of total sleep time. Other variables, including LaSO_2_, MaSO_2_, and TS90%, were also assessed. All PSG studies were scored according to the criteria of the American Academy of Sleep Medicine published in 2012 [24]. The severity of OSAHS was classified based on AHI status as follows: no OSAHS, < 5 events/h; mild OSAHS, 5 to < 15 events/h; moderate OSAHS, 15 to < 30 events/h; and severe OSAHS, ≥ 30 events/h.

### Assessment of NAFLD

Abdominal ultrasonography was performed by the trained sonographers using a Toshiba SSA-660A instrument (Toshiba, Tokyo, Japan) with a 2–5 MHz curved array probe. According to the revised 2018 NAFLD definition and treatment guidelines[25], diagnosis of fatty liver disease required the presence of at least two of the following three abnormal findings: diffusely increased liver near field ultrasound echo (‘bright liver’) and increased liver echotexture when compared to the kidneys, vascular blurring and deep attenuation of ultrasound signal. Participants were categorized into having NAFLD if they were diagnosed with fatty liver through ultrasonography and the weekly alcohol intake of < 210 g in men and < 140 g in women. In addition, other liver diseases (including chronic hepatitis B or C, operations on the liver, autoimmune liver diseases, cirrhotic or liver cancer, etc.) were identified by the results of their annual medical examinations (e.g., positive hepatitis B or C serology) and/or the self‐reported history of liver diseases.

### Statistical analysis

All statistical analyses were carried out with IBM SPSS Statistics for windows (Version 25.0 IBM Corp. Released 2017). Armonk, NY). Normally distributed data were expressed as means ± standard deviations, while skewed data were expressed as medians with interquartile range (IQR). Categorical data were expressed as proportions (percentage). Using the χ2 test for categorical data, the independent t-test for normally distributed data, and the Mann–Whitney U test for skewed data, the differences between groups with and without NAFLD were analyzed. Using Spearman's rank correlation coefficients, the relationships between platelet-related parameters and relevant factors were evaluated. An examination of the association between platelet-related characteristics and NAFLD, which served as the dependent variable, was accomplished by means of a binary logistic regression analysis. Through the use of receiver operating characteristic curve (ROC) analysis, the predictive value of PLR, WBC/MPV, and the combination of the two indices was investigated. P < 0.05 was deemed statistically significant for all two-tailed tests.

## Results

### Demographic data, anthropometric and polysomnographic variables in all participants

Our study comprised 351 OSAHS patients including 283 males and 68 females, with a mean age of 51.32 ± 13.65 years and a mean BMI of 28.2 ± 11.1 kg/m^2^. According to the ultrasound diagnosis of NAFLD, participants were allocated to OSAHS-only group (n = 72) and OSAHS + NAFLD group (n = 279). The anthropometric and polysomnographic characteristics of the patients were summarized in Table 1. We found that age, history of smoking, neck circumference (NC), waist circumference (WC) and BMI differed significantly between the groups (all *P* < 0.05), whereas gender, ESS score,and medical history, such as hypertension and diabetes did not. Furthermore, no significant differences were observed in polysomnographic parameters, including AHI, LaSO_2_, MaSO_2_, TS90% and ODI. However, the OSAHS + NAFLD group tended to have a higher AHI and a longer TS90% than the OSAHS-only group, suggesting that apnea and intermittent hypoxia were more severe in the OSAHS + NAFLD group.

**Table 1:** Comparison of anthropometric and polysomnographic parameters between the OSAHS-only and OSAHS+NAFLD group

	OSAHS-only	OSAHS + NAFLD	*P*
Subjects, n	72	279	
Age, years	55.79 ± 14.18	49.83 ± 13.13	0.002
Male sex, number (%)	54 (75.00)	229 (82.08)	0.175
Hypertension, number (%)	32 (44.44)	150 (53.76)	0.158
Diabetes, number (%)	11 (15.28)	53 (19.00)	0.466
Smoking, number (%)	18 (25.00)	108 (38.71)	0.031
NC (cm)	38.00 (35.00, 41.45)	40.00 (38.00, 42.00)	0.000
WC (cm)	93.50 (86.75, 101.00)	100.00 (94.00, 106.00)	0.000
BMI (kg/m^2^)	24.95 (23.23, 27.45)	27.55 (25.99, 29.87)	0.000
ESS score	7.00 (3.00, 12.50)	7.00 (4.00, 10.00)	0.279
AHI (events/h)	31.35 (17.80, 44.13)	34.00 (15.70, 56.93)	0.345
LaSO_2_ (%)	77.00 (62.25, 84.75)	77.00 (66.75, 83.00)	0.694
MaSO_2_ (%)	94.00 (91.25, 95.00)	94.00 (92.00, 95.00)	0.941
TS90% (min/h)	6.98 (1.45, 31.80)	10.60 (2.15, 45.15))	0.363
ODI (events/h)	24.20 (11.65, 40.65)	24.10 (10.20, 46.03)	0.612

Normally distributed data were expressed as mean ± SD, skewed data (including NC, WC, BMI, ESS score, AHI, LaSO_2_, MaSO_2_, TS90%, and ODI) were presented as median (interquartile range). Categorical variables were expressed as number (percentage). *NC* neck circumference, *WC* waist circumference, *BMI* Body mass index, *ESS score* Epworth Sleepiness Scale score, *AHI* apnea–hypopnea index, *LaSO*_*2*_ lowest O_2_ saturation, *MaSO*_*2*_ average O_2_ saturation, *TS90%* the percentage of total sleep time spent with SpO_2_ < 90%, *ODI* oxygen desaturation index

Normally distributed data were expressed as mean ± SD, skewed data (including NC, WC, BMI, ESS score, AHI, LaSO_2_, MaSO_2_, TS90%, and ODI) were presented as median (interquartile range). Categorical variables were expressed as number (percentage). *NC* neck circumference, *WC* waist circumference, *BMI* Body mass index, *ESS score* Epworth Sleepiness Scale score, *AHI* apnea–hypopnea index, *LaSO*_*2*_ lowest O_2_ saturation, *MaSO*_*2*_ average O_2_ saturation, *TS90%* the percentage of total sleep time spent with SpO_2_ < 90%, *ODI* oxygen desaturation index.

### Biochemical and hematological parameters in all participants

Biochemical and hematological parameters were reported in Table 2. There were no significant differences between two groups in terms of TC, TBIL, ALP or fasting glucose. However, OSAHS patients with NAFLD had significantly higher TG, ALT, AST, LDL-C, GGT, FLI and APRI than those without NAFLD (all *P* < 0.001), while had lower HDL-C levels than the OSAHS-only group (all *P* < 0.001). WBC/MPV, lymphocyte count, and white blood cell count were significant higher, while PLR was lower in OSAHS + NAFLD group. Nevertheless, there was a remarkable fact that no significant differences were observed in absolute value, such as PC, MPV, PDW, PCT and MPC (all *P* > 0.05). And the percentage of patients with upper limit of normal lymphocyte and white blood cell count was significant higher in OSAHS + NAFLD group than in OSAHS-only group (both *P* < 0.05), whereas there was no significant difference in the percentage of patients with upper limit of PC and MPV (both *P* > 0.05).

**Table 2:** Comparison of biochemical and hematological parameters between groups

	OSAHS-only	OSAHS + NAFLD	*P*
TG (mmol/L)	1.28 (0.84, 1.72)	1.91 (1.36, 2.62)	0.000
TC (mmol/L)	4.49 (3.73, 5.15)	4.63 (4.04, 5.20)	0.187
ALT (U/L)	21.00 (16.00, 28.00)	32.00 (21.00, 49.00)	0.000
AST (U/L)	19.00 (17.00, 22.00)	22.00 (18.00, 29.00)	0.000
LDL-C (mmol/L)	2.88 (2.14, 3.58)	3.00 (2.45, 3.72)	0.000
HDL-C (mmol/L)	1.15 (0.91, 1.38)	0.97 (0.86, 1.10)	0.000
TBIL (umol/L)	11.60 (8.60, 14.30)	11.20 (8.40, 13.80)	0.573
ALP (U/L)	64.00 (51.00, 78.00)	67.00 (55.00, 82.00)	0.265
GGT (U/L)	23.00 (18.00, 39.00)	36.00 (23.00, 55.00)	0.000
Fasting glucose (mmol/L)	4.92 (4.52, 5.41)	5.07 (4.59, 5.85)	0.149
PC (10^9/L)	221.00 (198.00, 256.00)	225.00 (194.00, 269.00)	0.526
Upper limit of normal PC, number (%)	1 (1.40)	8 (2.90)	0.772
MPV (fL)	8.90 (8.30, 9.50)	8.70 (7.90, 9.70)	0.774
Upper limit of normal MPV, number (%)	0 (0.00)	3 (1.10)	0.501
PDW (%)	49.20 (45.60, 57.70)	51.00 (46.30, 56.90)	0.396
PCT (%)	0.20 (0.17, 0.22)	0.20 (0.17, 0.23)	0.514
MPC (g/L)	252.00 (228.00, 276.00)	254.00 (233.00, 274.00)	0.460
PLR	126.92 (103.92, 148.00)	104.47 (84.76, 134.35)	0.000
WBC/MPV	0.64 (0.53, 0.78)	0.78 (0.64, 0.97)	0.000
Lymphocyte count. (10^9/L)	1.75 (1.51, 2.01)	2.18 (1.77, 2.60)	0.000
Upper limit of normal lymphocyte count, number (%)	0 (0.00)	14 (5.80)	0.045
White blood cell count (10^9/L)	5.76 (4.97, 6.65)	6.83 (5.79, 8.19)	0.000
Upper limit of normal white blood cell count, number (%)	2 (2.80)	33 (12.00)	0.037
FLI	1.04 (0.27, 2.01)	3.38 (1.52, 8.10)	0.000
APRI	0.22 (0.18, 0.26)	0.25 (0.20, 0.34)	0.001

Skewed data were presented as median (interquartile range). *TG* triglyceride, *TC* total cholesterol, *ALT* alanine transaminase, *AST* aspartate transaminase, *LDL-C* low-density lipoprotein cholesterol, *HDL-C* high-density lipoprotein cholesterol, *TBIL* total bilirubin, *ALP* alkaline phosphatase, *GGT* Gamma-glutamyl transferase, *PC* platelet count, *MPV* mean platelet volume, *PDW* platelet distribution width, *PCT* plateletcrit, *MPC* average concentration of platelet contents, *PLR* platelet to lymphocyte ratio, *WBC/MPV* white blood cell count to mean platelet volume ratio, *FLI* fatty liver index, *APRI* AST platelet ratio index

### Correlation analysis of platelet-related parameters with NAFLD in OSAHS patients

Table 3 revealed the correlations between PC, MPV, PLR, WBC/MPV, and other variables. PLR was negatively correlated with NAFLD (r = −0.250, *P* < 0.001), whereas WBC/MPV was positively correlated with NAFLD (r = 0.241, *P* < 0.001). In addition, PLR was positively correlated with MaSO_2_ (r = 0.115, *P* = 0.035), and WBC/MPV was positively correlated with ALT (r = 0.163, *P* = 0.006). Furthermore, platelet count, as expressions of platelet activation, had a negative correlation with LaSO_2_ (r = -0.107, *P* = 0.045), while MPV had a positive correlation with TS90% (r = 0.126, *P* = 0.019), suggesting sleep apnea and intermittent hypoxia played important roles in platelet activation.

**Table 3:** Spearman rank correlation coefficients between hematological parameters (platelet count, MPV, PLR and WBC/MPV) and NAFLD, metabolic, and polysomnographic characteristics

	PC		MPV		PLR		WBC/MPV	
	r	*P*	r	*P*	r	*P*	r	*P*
NAFLD	0.034	0.527	−0.015	0.774	−0.250	0.000	0.241	0.000
AHI	0.064	0.229	0.051	0.343	−0.054	0.314	0.075	0.162
TS90%	0.049	0.369	0.126	0.019	−0.040	0.461	0.009	0.868
LaSO_2_	−0.107	0.045	−0.053	0.321	−0.028	0.600	−0.049	0.362
MSaO_2_	0.031	0.564	−0.055	0.313	0.115	0.035	−0.057	0.292
ODI	0.045	0.409	0.073	0.178	−0.059	0.278	0.062	0.253
TG	0.105	0.050	0.060	0.266	0.011	0.850	0.060	0.322
ALT	0.147	0.006	−0.104	0.053	−0.036	0.552	0.163	0.006
AST	0.098	0.068	−0.036	0.503	0.002	0.906	0.075	0.209

*AHI* apnea–hypopnea index*, TS90%* the percentage of total sleep time spent with SpO_2_ < 90%, *LaSO*_*2*_ lowest O_2_ saturation, *MaSO*_*2*_ average oxygen saturation, *ODI* oxygen desaturation index, *TG* triglyceride, *ALT* alanine transaminase, *AST* aspartate transaminase

### Predictors of NAFLD in OSAHS patients according to logistic regression analysis

Logistic regression analysis highlighted WBC/MPV as a significant predictor of NAFLD in OSAHS populations with the highest odds ratio (OR) after adjustment, reaching 7.559 (95% CI: 1.411–40.486, *P* = 0.018), followed by gender (OR: 3.672; 95% CI: 1.231–10.954, *P* = 0.020), BMI (OR: 1.191; 95% CI: 1.028–1.380, *P* = 0.020), MaSO_2_ (OR: 1.134; 95% CI: 1.011–1.272, *P* = 0.031), ALT (OR: 1.049; 95% CI: 1.017–1.083, *P* = 0.003), HDL-C (OR: 0.070; 95% CI: 0.013–0.396, *P* = 0.0003), and PLR (OR: 0.980; 95% CI:0.970–0.991, *P* < 0.001)(Table 4).

**Table 4:** Risk factors for OSAHS with NAFLD: binary logistic regression analysis

Factors	OR	95%CI	*P*
Gender	3.672	(1.231, 10.954)	0.020
BMI	1.191	(1.028, 1.380)	0.020
MaSO_2_	1.134	(1.011, 1.272)	0.031
TG	1.384	(0.860, 2.226)	0.181
HDL-C	0.070	(0.013, 0.396)	0.003
ALT	1.049	(1.017, 1.083)	0.003
PLR	0.980	(0.970, 0.991)	0.000
WBC/MPV	7.559	(1.411, 40.486)	0.018

*BMI* body mass index, *MaSO*_*2*_ average oxygen saturation, *TG* triglyceride, *HDL-C* high-density lipoprotein cholesterol, *ALT* alanine transaminase, *PLR* platelet to lymphocyte ratio, *WBC/MPV* white blood cell count to mean platelet volume ratio

### Comparison of the parameters in the predictive power of NAFLD in OSAHS

**Fig. 1:**
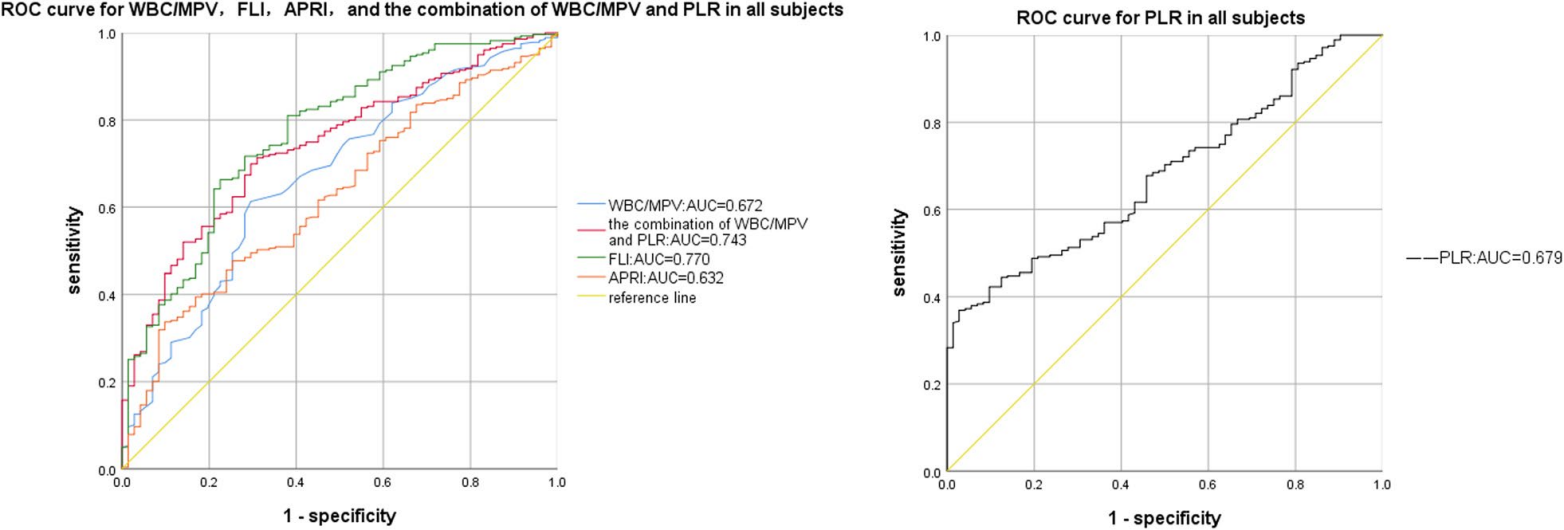
ROC curves and relat ed AUCs for WBC/MPV, PLR, FLI, APRI, and the combination of WBC/MPV and PLR in predicting the occurrence of NAFLD in all subjects. *AUC* area under curve, *BMI* body mass index, *PLR* platelet to lymphocyte ratio, *WBC/MPV* white blood cell count to mean platelet volume ratio, *FLI* fatty liver index, *APRI* AST platelet ratio index, *ROC* receiver operating characteristic

**Fig. 2:**
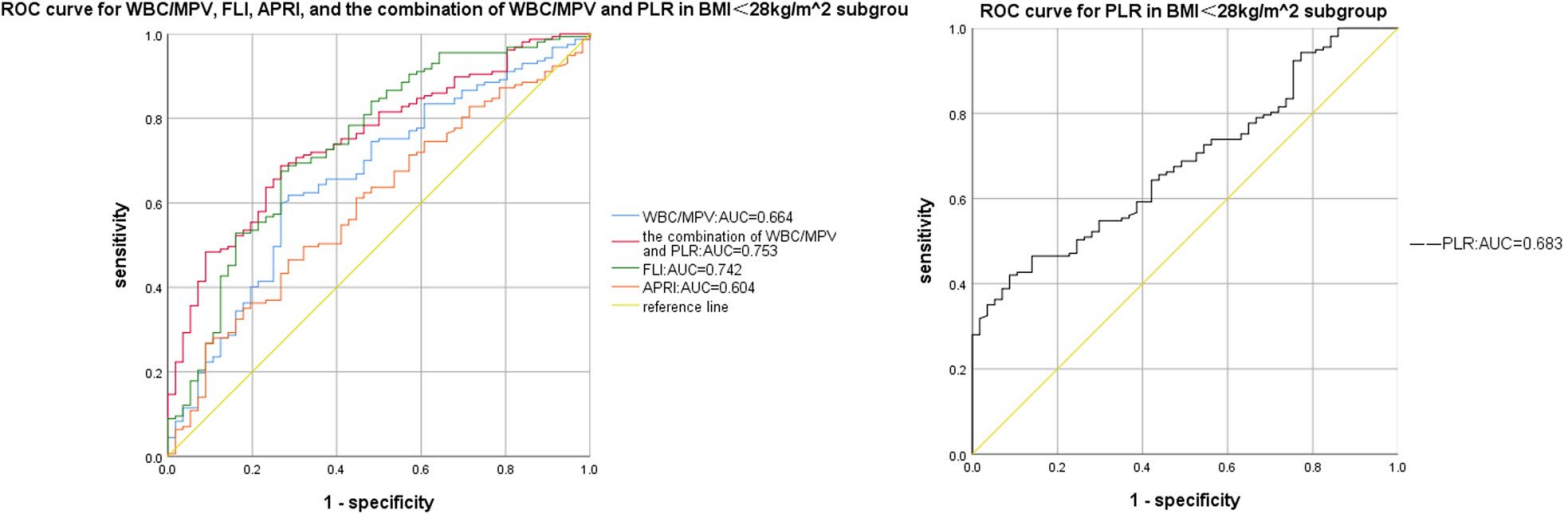
ROC curves and related AUCs for WBC/MPV, PLR, FLI, APRI, and the combination of WBC/MPV and PLR in predicting the occurrence of NAFLD in BMI < 28 kg/m^2^ subgroup. *AUC* area under curve, *BMI* body mass index, *PLR* platelet to lymphocyte ratio, *WBC/MPV* white blood cell count to mean platelet volume ratio, *FLI* fatty liver index, *APRI* AST platelet ratio index, *ROC* receiver operating characteristic

**Fig. 3:**
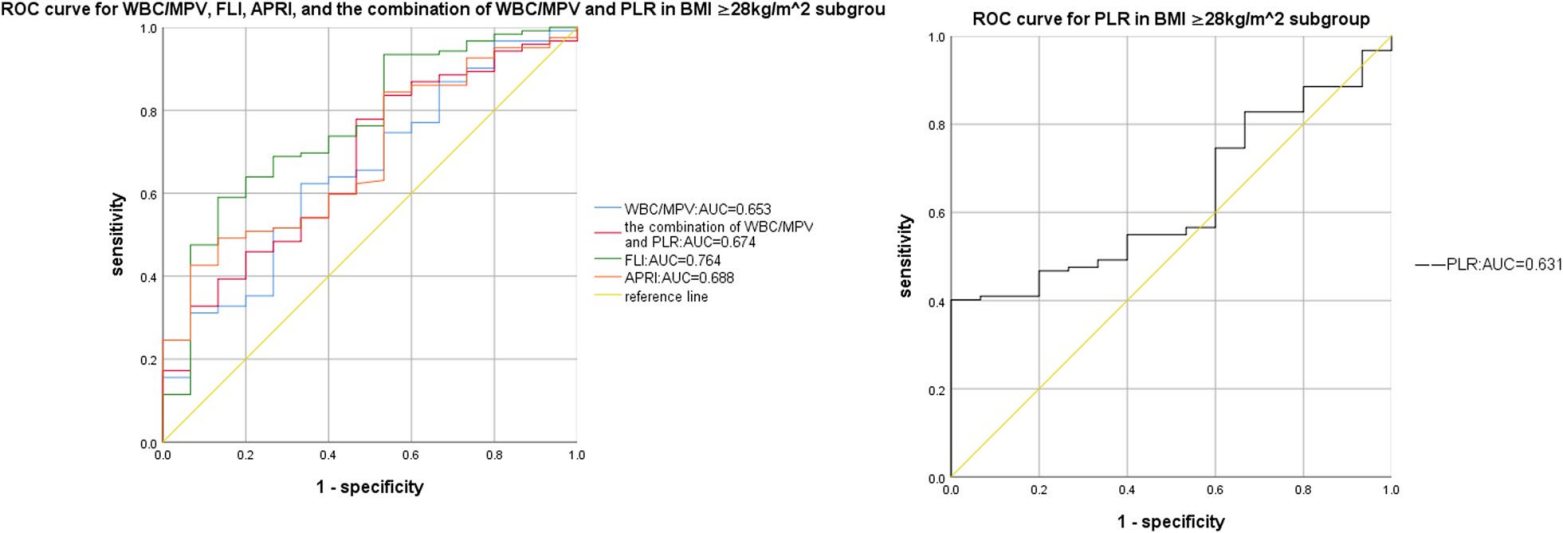
ROC curves and related AUCs for WBC/MPV, PLR, FLI, APRI, and the combination of WBC/MPV and PLR in predicting the occurrence of NAFLD in BMI ≥ 28 kg/m^2^ subgroup. *AUC* area under curve, *BMI* body mass index, *PLR* platelet to lymphocyte ratio, *WBC/MPV* white blood cell count to mean platelet volume ratio, *FLI* fatty liver index, *APRI* AST platelet ratio index, *ROC* receiver operating characteristic

**Fig. 4:**
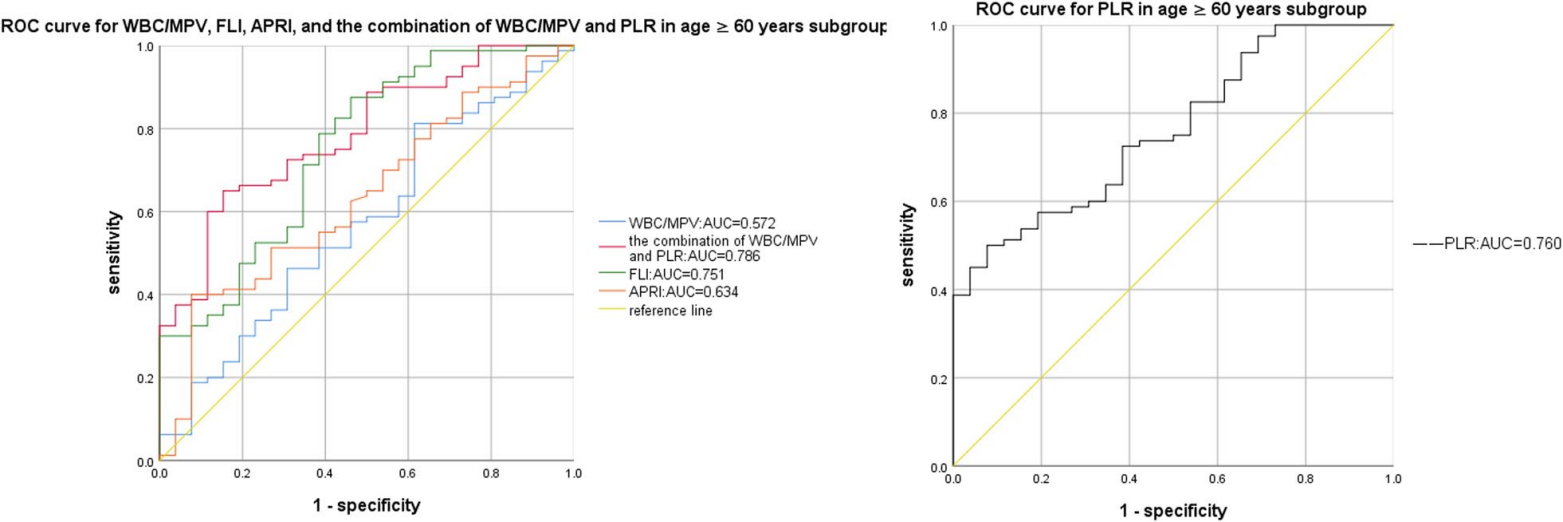
ROC curves and related AUCs for WBC/MPV, PLR, FLI, APRI, and the combination of WBC/MPV and PLR in predicting the occurrence of NAFLD in age < 60 years subgroup. *AUC* area under curve, *BMI* body mass index, *PLR* platelet to lymphocyte ratio, *WBC/MPV* white blood cell count to mean platelet volume ratio, *FLI* fatty liver index, *APRI* AST platelet ratio index, *ROC* receiver operating characteristic

**Fig. 5:**
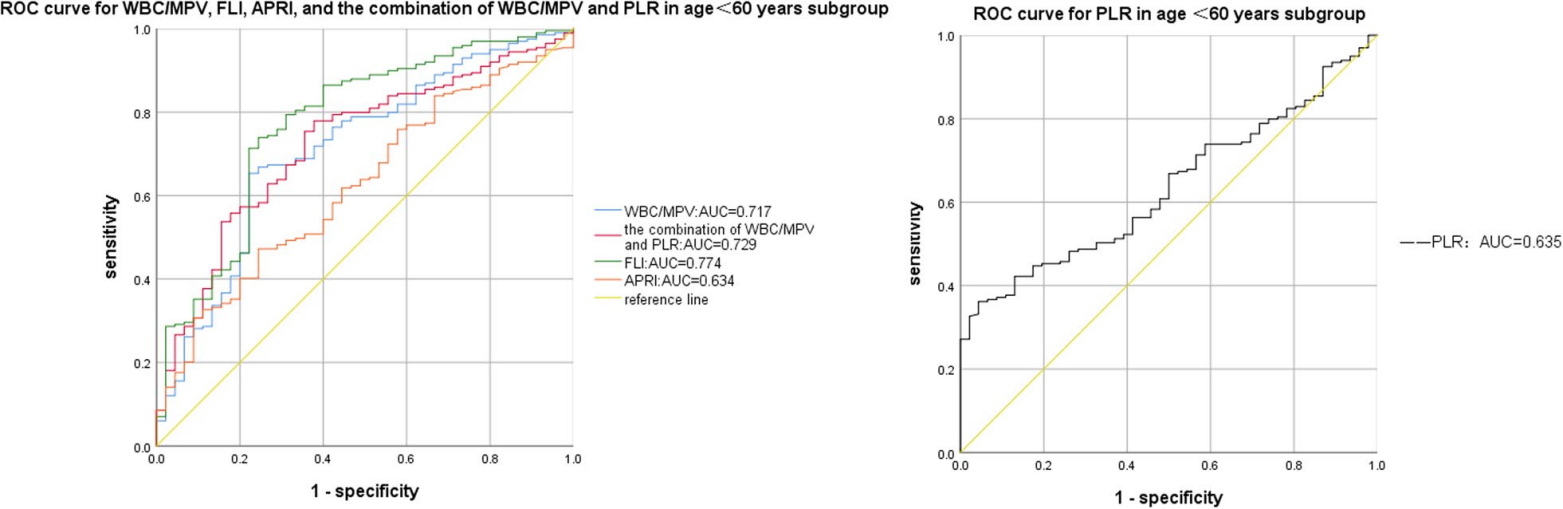
ROC curves and related AUCs for WBC/MPV, PLR, FLI, APRI, and the combination of WBC/MPV and PLR in predicting the occurrence of NAFLD in age ≥ 60 years subgroup. *AUC* area under curve, *BMI* body mass index, *PLR* platelet to lymphocyte ratio, *WBC/MPV* white blood cell count to mean platelet volume ratio, *FLI* fatty liver index, *APRI* AST platelet ratio index, *ROC* receiver operating characteristic

The results of the ROC curve analysis of the WBC/MPV, PLR, the combination of WBC/MPV and PLR, FLI, and APRI corresponding to 95% CI were shown in Table 5 and Figs. 1, 2, 3, 4 and 5. The AUC of NAFLD was the highest for FLI at 0.742 (95% CI 0.708–0.831), followed by the combination of WBC/MPV and PLR (0.743, 95% CI 0.684–0.801), PLR (0.679, 95% CI 0.618–0.740), WBC/MPV (0.664, 95% CI 0.602–0.741), and APRI (0.632, 95% CI 0.563–0.700) in all subjects. In the BMI subgroup analysis, the AUCs for the combination of WBC/MPV and PLR was 0.753 (95% CI 0.684–0.822), the highest in subjects with BMI < 28 kg/m^2^. In BMI ≥ 28 kg/m^2^ subjects, the AUC for the combination of WBC/MPV and PLR with NAFLD was 0.674 (95% CI 0.536–0.812), ranked only after FLI (0.764, 95% CI 0.636–0.892) and APRI (0.688, 95% CI 0.562–0.814). In the age subgroup analysis, the AUCs for the combination of WBC/MPV and PLR of age < 60 years old and age ≥ 60 years old with NAFLD were 0.729 (95% CI 0.652–0.806) and 0.786 (95% CI 0.692–0.880), which were the highest in age ≥ 60 years subgroup and the second highest in age < 60 years subgroup, respectively. Compared with the other four parameters, the most sensitive parameter for predicting NAFLD was the combination of WBC/MPV and PLR in BMI < 28 kg/m^2^ subgroup, BMI ≥ 28 kg/m^2^ subgroup, age < 60 years subgroup, and in all subjects.

**Table 5:** Areas under the receiver-operating characteristic curves for each parameter for predicting non-alcoholic fatty liver disease

Parameters	Area under the ROC curve	95% CI	*P*	SE	Cut off value	Sensitivity (%)	Specificity (%)
*All subjects (n* = *351)*							
WBC/MPV	0.672	(0.602, 0.741)	0.000	0.035	0.725	0.613	0.708
PLR	0.679	(0.618, 0.740)	0.000	0.031	91.225	0.369	0.972
The combination of WBC/MPV and PLR	0.743	(0.684, 0.801)	0.000	0.030	0.790	0.669	0.694
FLI	0.770	(0.708, 0.831)	0.000	0.031	2.101	0.663	0.775
APRI	0.632	(0.563, 0.700)	0.001	0.035	0.299	0.337	0.903
*BMI* < *28 kg/m*^*2*^* (n* = *214)*							
WBC/MPV	0.664	(0.582, 0.746)	0.000	0.042	0.720	0.618	0.719
PLR	0.683	(0.609, 0.756)	0.000	0.038	97.090	0.420	0.333
The combination of WBC/MPV and PLR	0.753	(0.684, 0.822)	0.000	0.035	0.733	0.688	0.736
FLI	0.742	(0.663, 0.820)	0.000	0.040	1.265	0.675	0.732
APRI	0.604	(0.520, 0.687)	0.020	0.043	0.259	0.465	0.184
*BMI* ≥ *28 kg/m*^*2*^* (n* = *137)*							
WBC/MPV	0.653	(0.511, 0.795)	0.054	0.072	0.726	0.623	0.667
PLR	0.631	(0.513, 0.748)	0.099	0.060	91.545	0.402	1.000
The combination of WBC/MPV and PLR	0.674	(0.536, 0.812)	0.028	0.070	0.859	0.779	0.533
FLI	0.764	(0.636, 0.892)	0.001	0.065	6.137	0.590	0.866
APRI	0.688	(0.562, 0.814)	0.018	0.064	0.290	0.426	0.933
*Age* < *60 years (n* = *245)*							
WBC/MPV	0.717	(0.634, 0.800)	0.000	0.042	0.726	0.653	0.783
PLR	0.635	(0.560, 0.710)	0.004	0.038	91.395	0.362	0.957
The combination of WBC/MPV and PLR	0.729	(0.652, 0.806)	0.000	0.039	0.760	0.779	0.630
FLI	0.774	(0.695, 0.853)	0.000	0.040	2.101	0.739	0.756
APRI	0.632	(0.548, 0.716)	0.005	0.043	0.261	0.472	0.761
*Age* ≥ *60 years (n* = *106)*							
WBC/MPV	0.572	(0.445, 0.669)	0.271	0.065	0.591	0.813	0.385
PLR	0.760	(0.663, 0.857)	0.000	0.050	102.380	0.500	0.923
The combination of WBC/MPV and PLR	0.786	(0.692, 0.880)	0.000	0.048	0.822	0.650	0.846
FLI	0.751	(0.640, 0.862)	0.000	0.057	0.690	0.875	0.538
APRI	0.634	(0.515, 0.753)	0.040	0.061	0.289	0.400	0.923

*PLR* platelet to lymphocyte ratio, *WBC/MPV* white blood cell count to mean platelet volume ratio, *FLI* fatty liver index, *APRI* AST platelet ratio index, *CI* Confidence interval, *SE* Standard error

## Discussion

In this study, we evaluated platelet-related biomarkers as potential predictors of NAFLD among OSAHS participants. Logistic regression analysis identified WBC/MPV and MaSO_2_ as risk factors, while PLR as a protective factor for NAFLD in OSAHS populations. In addition, a combination of PLR and WBC/MPV showed a good prediction capacity regarding NAFLD compared with WBC/MPV, PLR, FLI, and APRI. Further subgroup ROC analysis indicated a combination of PLR and WBC/MPV presented the largest AUC for the detection of NAFLD in BMI < 28 kg/m^2^ subgroup (0.753, 95% CI 0.684–0.822), and in the age ≥ 60 years subgroup (0.786, 95% CI 0.692–0.880).

NAFLD has become more and more common worldwide, especially in the OSAHS populations, and it can result in a range of serious complications, including liver failure and even death [26], so if we can identify NAFLD as early as possible, and then carry out the preventive intervention, the mortality can be significantly reduced. Clinically, liver biopsy is the gold standard for screening NAFLD [27]. However, the invasiveness and high cost of liver biopsy have limited its application [28, 29]. In recent years, the relation between platelet-related parameters and liver diseases has caught the interest of many scientists since platelet-related parameters can represent the inflammatory state of the body and even provide the advantages of simplicity, cost-effectiveness, non-invasiveness, etc. [11–13].

Our study showed that AHI and TS90% were higher in the OSAHS + NAFLD group. Subsequent binary logistic regression analysis found that MaSO_2_ was an independent risk factor for NAFLD, suggesting that nocturnal hypoxia played an important role in the development of NAFLD in OSAHS participants. According to previous studies, nocturnal hypoxia was linked with systemic inflammation. He et al. [30] developed an IH 3T3-L1 adipocyte and rat model respectively, recapitulating the nocturnal oxygen profile in OSAHS. The findings demonstrated that NF-κB DNA binding activities were positively correlated with the severity of OSAHS in cellular models. Additionally, the mRNA and protein levels of TNF- and IL-6 increased with the severity of OSAHS in both cellular and animal models. Many researchers have demonstrated that the activation of inflammatory and apoptotic factors caused by nocturnal hypoxia contributes to the pathogenesis and progression of NAFLD in OSAHS individuals [31]. Polotsky et al. [32] studied 90 consecutive bariatric patients who underwent PSG, and reported that nocturnal oxygen desaturation might predispose them to hepatic inflammation, hepatocyte ballooning, and liver fibrosis. A series of researches have reported that liver injuries are initiated by histology-derived oxidative stress, with the initial inflammatory signaling cascade culminating in a positive feedback loop for oxidative stress and the activation of various inflammatory mediators, such as NF-κB, TNF-α, HIF-1a, etc., causing liver tissue microcirculation dysfunction and systemic inflammatory responses [33–35].

Platelets play a crucial role in liver injury and liver fibrosis. Ghafoory et al. [36] demonstrated that activated platelets were found during acute liver injury (6 h) in mice exposed to CCl4, and mice with temporary platelet depletion were partially protected against CCl4-induced fibrosis, indicating a link between platelet activation and fibrosis. Notably, platelets can cause sinusoidal endothelial cells to emit a significant number of chemokines and increase the migration of neutrophils and lymphocytes [37], which ultimately induces liver injury and fibrosis, indicating that an index representing platelets and inflammation may play a role in predicting NAFLD.

PLR, a newly discovered sensitive index of the inflammation system in the body, is able to reflect the state of inflammation [38]. PLR seemed to be mostly explored in chronic HBV/HCV and HCC patients, and prior research revealed that PLR levels were higher in those with more advanced HCC and a higher recurrence risk [14], while low levels of PLR were observed in chronic HBV/HCV patients [15]. To the best of our knowledge, this is the first study that presented the association between PLR and the occurrence of NAFLD in OSASH patients, even after adjusted for well-known risk factor for NAFLD in logistic regression analysis. In this study, PLR levels of OSAHS complicated in NAFLD group were lower than those of the OSAHS-only group, and PLR seemed to be a reliable predictor of NAFLD in the OSAHS population. Given that PLR is a composite indicator that comprises both platelets and lymphocytes, the activation and aggregation of platelets, as well as inflammation and immune responses in the body, may all play a part in the development of NAFLD in OSAHS patients. In addition, PLR was shown to be positively connected with MaSO_2_, implying that PLR is linked to hypoxia in this research. Prior studies have shown that oxyhemoglobin desaturation is associated with platelet activation [39–41], and that the severity of desaturation in OSAHS patients is positively associated with excess catecholamine levels [42]. Furthermore, it is known that increased shear stress owing to sympathetic nervous system activation and high blood pressure activates platelets, hence boosting inflammatory responses in numerous target organs, including the liver [42].

WBC/MPV is usually used to predict thrombosis-related events, particularly in cardiovascular, cerebrovascular, and peripheral vascular diseases. There hasn't been any research done on the link between WBC/MPV, OSAHS, and NAFLD. We used WBC/MPV to predict NAFLD in OSAHS patients in a novel way since both OSAHS and NAFLD can lead to pathological changes in the blood system. Our research indicated that WBC/MPV ratio was greater in the OSAHS + NAFLD group, and the WBC/MPV ratio was an independent risk factor for OSAHS complicated with NAFLD. According to previous studies, patients with NAFLD exhibited higher white blood cell and platelet counts. The increased expression of inflammatory markers in NAFLD patients may promote thrombopoietin development, resulting in larger platelets in the bone marrow and platelet activation, which in turn causes liver damage, and creates a vicious cycle. In our research, we found that a combination of WBC/MPV and PLR can better predict the occurrence of NAFLD compared to PLR or WBC/MPV alone.

In this study, we also compared the efficacy of PLR, WBC/MPV, FLI, APRI, and the combination of WBC/MPV and PLR to predict the occurrence of NAFLD in patients with OSAHS. FLI and APRI were non-invasive scores widely used in the prediction of NAFLD [21, 22]. And our study revealed that the combination of WBC/MPV and PLR had a good prediction capacity in NAFLD, only ranked after FLI in all subjects. Further subgroup analysis presented a fact that the combination of WBC/MPV and PLR had an even better performance in NAFLD prediction in BMI < 28 kg/m^2^, and in the age ≥ 60 years subgroup. To be emphasized, there was no significant differences in the absolute value of platelet count and MPV. However, PLR and WBC/MPV, the composite indicator, have better performances in predicting NAFLD. We hypothesized that stress, changes in blood volume in vivo, and changes in specimen fluid volume during testing operations may affect absolute values of platelet count, lymphocyte count, white blood cell, and MPV, resulting in improved sensitivity and resistance to interference when using composite indicators for disease prediction.

There are a few problems with this study. As a matter of fact, our study only included a limited number of patients, and each of those patients was only evaluated at a single location. Therefore, we were unable to avoid selection bias when gathering data. Second, the PLR and WBC/MPV ratios were evaluated using a single measurement at the time of admission for the preliminary diagnosis. In order to avoid interference brought on by measurement mistakes in the subsequent study, further blood procedures will be needed to carry out multiple times at a variety of time points. Third, because a liver biopsy was not possible, a diagnosis of fatty liver was made using liver ultrasonography rather than a biopsy of the liver. Despite the fact that a previous study found that ultrasonography had a sensitivity of 89% and a specificity of 93% for NAFLD, and that it was widely used in population-based studies because of its non-invasiveness and accessibility, ultrasound has limited sensitivity in individuals with an elevated BMI, and it does not reliably detect mild steatosis [43]. Future investigations utilizing more precise methods, such as liver biopsy, would be required to confirm the findings of this study. Even with the aforementioned limitations, our study is the first cohort study to explore the diagnostic value of PLR and WBC/MPV in NAFLD among OSAHS patients, providing a non-invasive, cost-efficient and convenient method in the early-detection of NAFLD.

## Conclusion

PLR and WBC/MPV are closely related to liver inflammation in patients with OSAHS. PLR and WBC/MPC are potential risk factors for NAFLD. Furthermore, our findings highlighted the combination of PLR and WBC/MPV as an effective indicator of NAFLD in patients with OSAHS. Given the accessibility and simplicity of calculation, these indices could be widely used for screening NAFLD in individuals with OSAHS in clinical settings.

## Data Availability

The datasets used and analyzed during the current study are available from the corresponding author on reasonable request.

## Abbreviations

NAFLD: Non-alcoholic fatty liver disease
PLR: Platelet to lymphocyte count ratio
WBC/MPV: White blood cell count to mean platelet volume ratio
OSAHS: Obstructive sleep apnea–hypopnea syndrome
ROC curve: The receiver operating characteristic curve
MaSO_2_: Average oxygen saturation
NAFL: Non-alcoholic fatty liver
NASH: Non-alcoholic steatohepatitis
Liver/spleen CT: Liver/spleen computed tomography
MRI: Magnetic resonance imaging
ESS: Epworth sleepiness scale
TG: Triglyceride
TC: Total cholesterol
PC: Platelet count
MPV: Mean platelet volume
PDW: Platelet distribution width
PCT: Plateletcrit
MPC: Average concentration of platelet contents
IQR: Interquartile range
NC: Neck circumference
WC: Waist circumference
BMI: Body mass index
AHI: Apnea–hypopnea index
LaSO_2_: Lowest O_2_ saturation
MaSO_2_: Average O_2_ saturation
TS90%: The percentage of total sleep time spent with SpO_2_ < 90%,
ODI: Oxygen desaturation index
AST: Aspartate transaminase
LDL-C: Low-density lipoprotein cholesterol
HDL-C: High-density lipoprotein cholesterol
TBIL: Total bilirubin
ALP: Alkaline phosphatase
GGT: Gamma-glutamyl transferase
OR: Odds ratio
AUC: Area under the ROC curve
FLI: Fatty liver index
APRI: Aspartate aminotransferase to platelet ratio index
